# Amide Proton Transfer Weighted and Intravoxel Incoherent Motion Imaging in Evaluation of Prognostic Factors for Rectal Adenocarcinoma

**DOI:** 10.3389/fonc.2021.783544

**Published:** 2022-01-03

**Authors:** Juan Li, Liangjie Lin, Xuemei Gao, Shenglei Li, Jingliang Cheng

**Affiliations:** ^1^ Department of Magnetic Resonance Imaging (MRI), The First Affiliated Hospital of Zhengzhou University, Zhengzhou, China; ^2^ Advanced Technical Support, Philips Healthcare, Beijing, China; ^3^ Department of Pathology, The First Affiliated Hospital of Zhengzhou University, Zhengzhou, China

**Keywords:** APT, IVIM, rectal neoplasms, adenocarcinoma, magnetic resonance imaging

## Abstract

**Objectives:**

To analyze the value of amide proton transfer (APT) weighted and intravoxel incoherent motion (IVIM) imaging in evaluation of prognostic factors for rectal adenocarcinoma, compared with diffusion weighted imaging (DWI).

**Materials and Methods:**

Preoperative pelvic MRI data of 110 patients with surgical pathologically confirmed diagnosis of rectal adenocarcinoma were retrospectively evaluated. All patients underwent high-resolution T_2_-weighted imaging (T_2_WI), APT, IVIM, and DWI. Parameters including APT signal intensity (APT SI), pure diffusion coefficient (D), pseudo-diffusion coefficient (D*), perfusion fraction (f), and apparent diffusion coefficient (ADC) were measured in different histopathologic types, grades, stages, and structure invasion statuses. Receiver operating characteristic (ROC) curves were used to evaluate the diagnostic efficacy, and the corresponding area under the curves (AUCs) were calculated.

**Results:**

APT SI, D and ADC values of rectal mucinous adenocarcinoma (MC) were significantly higher than those of rectal common adenocarcinoma (AC) ([3.192 ± 0.661%] *vs*. [2.333 ± 0.471%], [1.153 ± 0.238×10^-3^ mm^2^/s] *vs*. [0.792 ± 0.173×10^-3^ mm^2^/s], and [1.535 ± 0.203×10^-3^ mm^2^/s] *vs*. [0.986 ± 0.124×10^-3^ mm^2^/s], respectively; all *P*<0.001). In AC group, the APT SI and D values showed significant differences between low- and high-grade tumors ([2.226 ± 0.347%] *vs*. [2.668 ± 0.638%], and [0.842 ± 0.148×10^-3^ mm^2^/s] *vs*. [0.777 ± 0.178×10^-3^ mm^2^/s], respectively, both *P*<0.05). The D value had significant difference between positive and negative extramural vascular invasion (EMVI) tumors ([0.771 ± 0.175×10^-3^ mm^2^/s] *vs*. [0.858 ± 0.151×10^-3^ mm^2^/s], *P*<0.05). No significant difference of APT SI, D, D*, f or ADC was observed in different T stages, N stages, perineural and lymphovascular invasions (all *P*>0.05). The ROC curves showed that the AUCs of APT SI, D and ADC values for distinguishing MC from AC were 0.921, 0.893 and 0.995, respectively. The AUCs of APT SI and D values in distinguishing low- from high-grade AC were 0.737 and 0.663, respectively. The AUC of the D value for evaluating EMVI involvement was 0.646.

**Conclusion:**

APT and IVIM were helpful to assess the prognostic factors related to rectal adenocarcinoma, including histopathological type, tumor grade and the EMVI status.

## Introduction

Colorectal cancer is a common malignancy of the digestive system, 30-35% are occurred in the rectum, and 90% are classified as adenocarcinoma (1, 2). Many factors are associated with therapeutic schedule and prognosis of rectal cancer, including tumor location, histological type, tumor grade, T stage, N stage, and related imaging indicators based on MRI, such as circumferential resection margin (CRM), and extramural vascular invasion (EMVI) statuses (3, 4). Rectal mucinous adenocarcinoma (MC) is a common subtype of rectal adenocarcinomas, which has a poor prognosis, and it is not sensitive to neoadjuvant chemoradiotherapy (5, 6). The selection of individualized treatment options for rectal cancer is based on accurate imaging evaluation.

Magnetic resonance imaging (MRI) is the most accurate test for preoperative assessment of rectal cancer. Conventional high-resolution MRI imaging, especially the small-field-of-view and thin-layer T_2_ weighted imaging (T_2_WI), not only clearly distinguishes the various layers of the rectal wall, but also displays the mesorectal fascia and EMVI (7, 8). Functional MRI has become increasingly widespread in recent years. Diffusion weighted imaging (DWI) is an example of functional MRI that reflects changes in tissue microenvironments by measuring the diffusion of water molecules in tissues. It has been applied in tumor TN stage, grading, and prognosis of rectal cancer in previous studies. However, the results were lack of consistency (9). Zhu et al. found the ADC values of low-grade adenocarcinoma were higher than those of high-grade adenocarcinoma, but the difference was not statistically significant (10). Several new MRI techniques have been used to evaluate the pathological features of rectal cancer, including intravoxel incoherent motion (IVIM), diffusion kurtosis imaging (DKI), and dynamic contrast-enhanced (DCE) imaging (11, 12). IVIM provides diffusion and perfusion information within tissue through the biexponential modelling of images acquired by multiple b values (13). Previous studies showed the ability of IVIM for the differential diagnosis of malignant and benign tumors, as well as reflect the biological behavior and predict prognosis (14–16). Amide proton transfer (APT) weighted imaging is a noninvasive molecular imaging technique based on chemical exchange saturation transfer (CEST). It measures the endogenous moving proteins and peptides by detecting the reduction in bulk water intensity, which indirectly reflects changes of the internal metabolism (17, 18). APT weighted imaging has been applied in studies of various cancers, it exhibits an excellent ability in tumor differentiation, grading, and discrimination of treatment related necrosis from recurrence (19–21). Li et al. suggested the utility of APT and IVIM may be a useful technique in the diagnosis and predicting the differentiation of squamous cell carcinoma (22). Jia et al. found a prediction model incorporating APT and IVIM in the tumor may be useful for predicting the response of hepatocellular carcinoma (HCC) to transarterial chemoembolization (TACE) pretreatment (23). There are few studies using APT on research of rectal cancer. Nishie et al. observed APT weighted imaging can predict the tumor response to neoadjuvant chemotherapy in patients with locally advanced rectal cancer (24). Previous studies have reported that tumors with high-grade, more advanced T stage, and lymph node metastasis had higher APT signal intensity (APT SI) (25–27). However, the previous studies were commonly with limited sample sizes, without involvement of the histopathologic type, perineural invasion and lymphovascular invasion, and without comparison to IVIM.

This study aims to investigate the ability of APT and IVIM in evaluation of prognostic factors for rectal adenocarcinoma, thereby to evaluate its reference value for assessing the malignant degree and predicting tumor aggressiveness, compared with results by conventional DWI.

## Materials and Methods

### Participants

**Figure 1:**
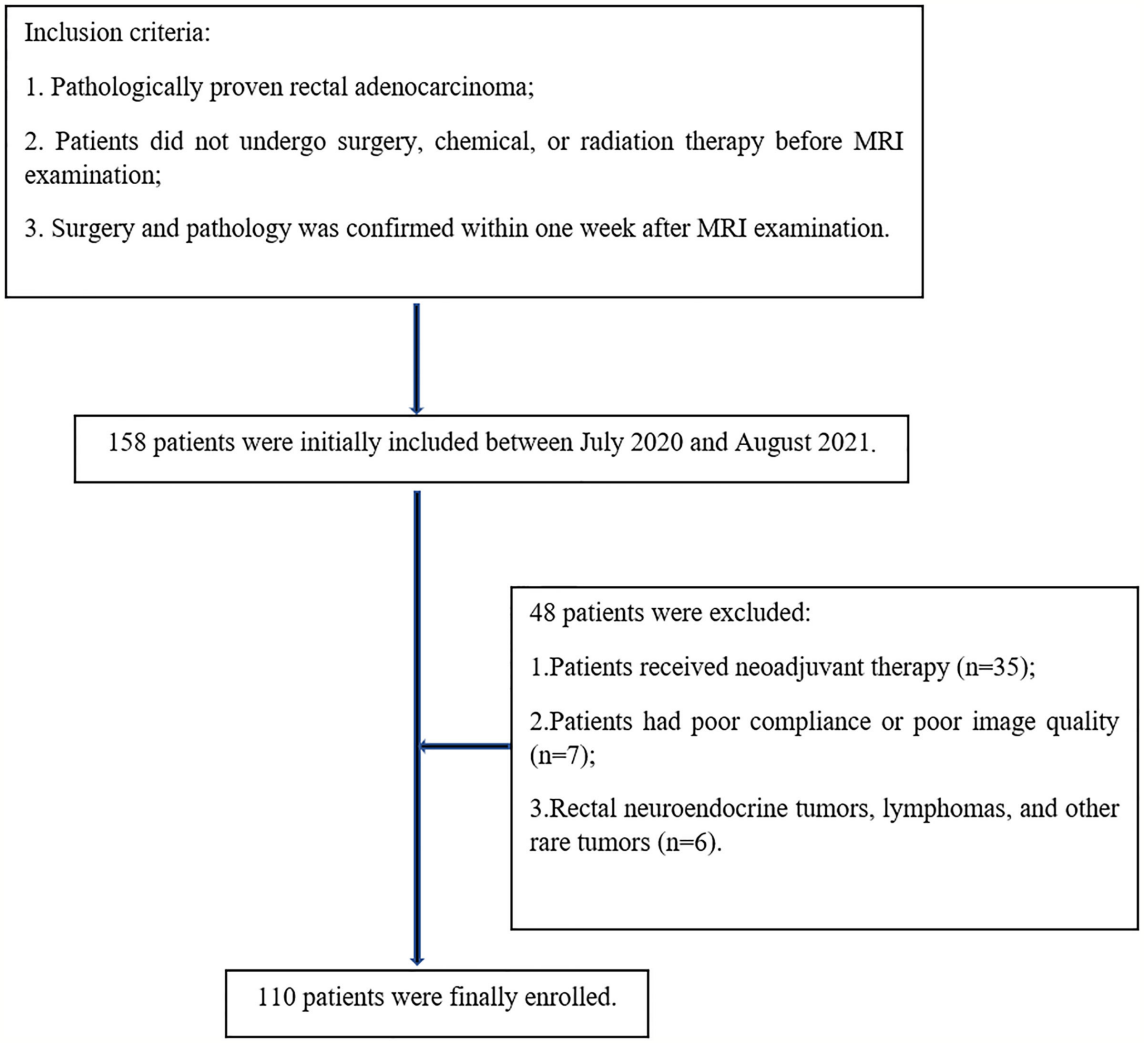
Flowchart of patient selection.

Preoperative pelvic MRI data of 158 patients with pathologically confirmed rectal cancer at our hospital were collected between July 2020 and August 2021. The inclusion criteria were as follows: pathologically proven rectal adenocarcinoma; patients did not undergo surgery, chemical, or radiation therapy before MRI examination; surgery and pathology was confirmed within one week after MRI examination. The exclusion criteria were as follows: patients received neoadjuvant therapy (n=35); patients had poor compliance or poor image quality (n=7); rectal neuroendocrine tumor, lymphomas, and other rare tumors (n=6). Finally, 110 patients were enrolled in this study (**Figure 1**).

### MR Imaging Protocols

MRI were performed on a 3T scanner (Ingenia CX, Philips Healthcare, Best, the Netherlands) with a 32-channel phase array coil. Patients were instructed to empty the rectum before examination. To suppress intestinal movement artifacts, 20 mg raceanisodamine hydrochloride injection (Suicheng Pharmaceutical Co, Ltd.) was given intramuscularly 5-10 min before examination. The scanning sequences included T_2_WI, T_1_-weighted imaging (T_1_WI), DWI, APT, IVIM, and dynamic contrast-enhanced T_1_WI. The oblique axial was positioned perpendicular to the long axis of the lesion. Detailed parameters for the sequences were listed in **Table 1**.

**Table 1:** MRI acquisition parameters.

Parameters	APT	IVIM	DWI	T_2_WI
Sequence	TSE	EPI	EPI	TSE
TR/TE (ms)	6540/8.3	4888/90	4421/77	3000/100
Field of view (mm^2^)	230×181	240×240	200×129	180×180
Slice thickness (mm)	5	5	4	4
No. of slices	9	24	24	24
Matrix	116×90	72×67	80×52	300×235
Spatial resolution (mm^3^)	2×2×5	3.3×3.58×5	2.5×2.46×4	0.6×0.7×4
b-values (s/mm^2^)	N/A	0,10,20,50,100,200,400,800,1200	0,800	N/A
Bandwidth (Hz/pixel)	647.2	2977.8	2365.9	254.1
TSE factor	174	N/A	N/A	15
Fat suppression	Yes	No	No	N0
Acquisition time	6min0s	4min53s	2min26s	2min54s

T_2_WI, T_2_-weighted imaging; TSE, turbo spin echo; EPI, echo-planar imaging; TR, repetition time; TE, echo time; N/A, not applicable.

High-resolution T_2_WI was helpful for estimating tumor location, the relationship with peritoneal reflection, tumor size, bowel circumferential involvement, CRM, and EMVI statuses. APT weighted images were acquired by using a 3D turbo spin echo (TSE) sequence for optimized signal-to-noise ratio. The continuous RF saturation for a duration of 2 seconds (each RF coil was turned on and off for 500 msec to generate four block RF pulses at 2 μT amplitude) (20). For convenience, the water frequency (around 4.75 ppm in the proton MR spectrum) is placed at 0 ppm of the Z-spectrum, in which the water signal saturation is measured as a function of saturation frequency. Data were acquired with seven different saturation frequency offsets with respect to the water resonance ( ± 3.5, ± 3.42, ± 3.58, −1560 ppm). A B_0_ map was derived from three echo acquisitions at +3.5 ppm for B_0_ correction (28). IVIM (with b values of 0, 10, 20, 50, 100, 200, 400, 800, 1200 s/mm^2^) were performed in the oblique axial plane using a single-shot echo planar imaging (ss-EPI) sequence with comparable parameters. The diffusion gradients were applied simultaneously along with three orthogonal directions. DWI (with b values of 0, 800 s/mm^2^) was also performed using the ss-EPI sequence.

### Data Processing and Analysis

APT weighted images were automatically generated on the console at the time of scan completion. After MR scans, all images were uploaded to the IntelliSpace Portal (ISP v10, Philips Healthcare) workstation for post processing or quantitative measurements. The MTR_asym_ (magnetization transfer ratio asymmetry) value at the frequency offset of +3.5 ppm was displayed as percent level (relative to S_0_) in the final APT images, and referred as APT SI:


APT SI=MTRasym [Δω=+3.5ppm] (%)

The IVIM data were processed by the application of advanced diffusion analysis (ADA) on the workstation with maps of the pure diffusion coefficient (D), pseudo-diffusion coefficient (D*) and perfusion fraction (f) generated. The linear fitting equation is as follows:


$${\text{S}}_{\text{b}}/{\text{S}}_{0}=(1-f)\exp(-b\times D)+f\;\exp(-b\times D\;*)$$

where S_b_ is the MR signal intensity with diffusion gradient; S_0_ is the MR signal intensity without diffusion gradient. The ADC maps were generated immediately after DWI data acquisition.

MRI images were analyzed by two radiologists experienced in gastrointestinal diseases diagnosis. Regions of interest (ROIs) on APT SI, D, D*, f and ADC images were manually selected for analysis, according to T_2_WI and pathology results. The ROIs of APT SI were drawn on APT-T2 merged images, then the same ROIs were copied to the ADC images for measuring values. The ROIs of D were drawn on D images, then the same ROIs were showed on the D* and f images for quantitative measurements. The ROIs were drawn at the level of the maximum extent of the tumor and the levels above and below it, and the averaged values were taken. Necrotic, cystic, and hemorrhagic regions were avoided.

### Pathologic Analyses

Pathological reports of rectal cancer were referred to standardized templates, including surgical procedures, gross and histological types, tumor grade, pathological stage, perineural invasion, lymphovascular invasion, cut edge infringement, and immunohistochemistry. According to world health organization (WHO) grading criteria, rectal common adenocarcinoma (AC) was classified as grade 1 (G1, well differentiated, >95% gland forming), grade 2 (G2, moderately differentiated, 50-95% gland forming), or grade 3 (G3, poorly differentiated, 0-49% gland forming). According to two-tiered grading system of WHO criteria, G1 and G2 tumors were classified as low-grade tumors, G3 tumors were classified as high-grade tumors. The staging criteria were evaluated according to the American Joint Committee on Cancer (AJCC) 8th edition. T staging was classified as pT1-2 and pT3-4 stage based on depth of tumor invasion. pT1-2 stage cancer was defined as disease confined to the muscularis propria, including pT1 and pT2 stage, and pT3-4 stage cancer was defined as disease extending beyond the muscularis propria, including pT3 and pT4 stage. Lymph node staging was performed based on results of postoperative pathology including pN0 stage: lack of regional lymph node metastasis, pN1 stage: less than 3 regional lymph node metastasis, and pN2 stage: 4 or more regional lymph node metastasis. Perineural invasion, lymphovascular invasion, and EMVI statuses were classified into positive and negative groups.

### Statistical Analysis

SPSS 22.0 software (IBM, Armonk, NY) was used for statistical analysis. The Kolmogorov-Smirnov test was performed for analyzing normality. Data conforming to the normal distribution were expressed as mean ± standard deviation (SD). The intraclass correlation coefficient (ICC) was used to evaluate the interobserver consistency of the measured parameters. ICC values of less than 0.40, 0.41–0.75, and greater than 0.75 were considered to indicate poor, fair, and good agreement, respectively. The t-test for independent samples was used to compare APT SI, D, D*, f and ADC parameters between pathological types (MC *vs*. AC), WHO grades (low- *vs*. high-grade), pT stages (pT1-2 *vs*. pT3-4), pN stages (pN1-2 *vs*. pN0), perineural invasion (positive *vs*. negative), lymphovascular invasion (positive *vs*. negative), and EMVI statuses (positive *vs*. negative). For parameters with significant differences between groups, the receiver operating characteristic (ROC) curve was used to analyze their diagnostic efficacy using the software of MedCalc v. 20.0 (MedCalc Software, Ostend, Belgium). DeLong test was used to compare the differences of area under ROC curves (AUCs). The forward model of binary logistic regression was applied for parameter fusion. Differences with *P*<0.05 were considered statistically significant.

## Results

### The Pathological Results of Rectal Adenocarcinomas

**Figure 2:**
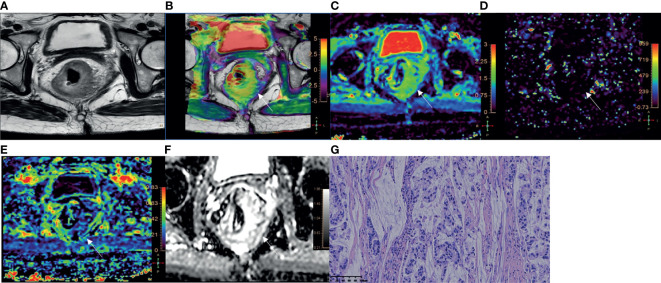
A 51-year-old female with MC. **(A)** Oblique axial T_2_-weighted image showed a mass with high intensity in the rectum. **(B)** APT-T2 merged image showed the mass with a mean APT SI of 3.4%. **(C–E)** D, D* and f maps showed the mass with values of 1.42×10^-3^ mm^2^/s, 5.00×10^-3^ mm^2^/s and 0.22, respectively. **(F)** The mass showed high intensity (1.87×10^-3^mm^2^/s) on the ADC map. **(G)** HE staining revealed mucinous adenocarcinoma. (×200).

**Figure 3:**
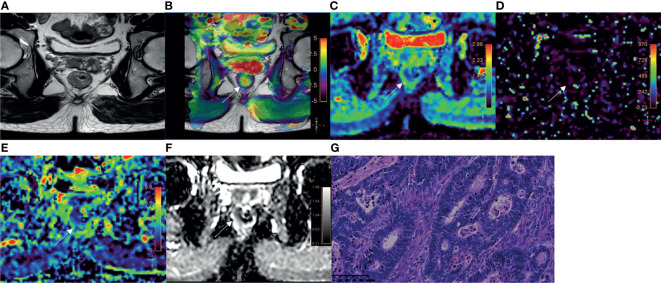
A 52-year-old male with AC of grade 2. **(A)** Oblique axial T_2_-weighted image showed a mass with slightly high intensity in the rectum. **(B)** APT-T2 merged image showed the mass with a mean APT SI of 2.1%. **(C–E)** D, D* and f maps showed the mass with values of 0.81×10^-3^ mm^2^/s, 7.74×10^-3^ mm^2^/s and 0.19, respectively. **(F)** The mass showed low intensity (1.03×10^-3^ mm^2^/s) on the ADC map. **(G)** HE staining revealed moderately differentiated adenocarcinoma. (×200).

**Figure 4:**
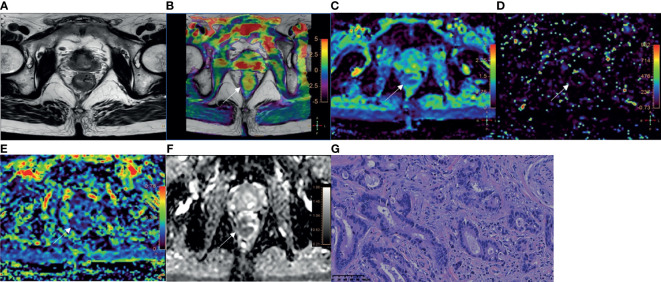
A 78-year-old male with AC of grade 3. **(A)** Oblique axial T_2_-weighted image showed a mass with slightly high intensity in the rectum. **(B)** APT-T2 merged image showed the mass with a mean APT SI of 2.8%. **(C–E)** D, D* and f maps showed the mass with values of 0.79×10^-3^mm^2^/s, 9.40×10^-3^mm^2^/s and 0.17, respectively. **(F)** The mass showed low intensity (0.93×10^-3^mm^2^/s) on the ADC map. **(G)** HE staining revealed poorly differentiated adenocarcinoma. (×200).

Among 110 rectal adenocarcinomas, 17 cases were MC and 93 cases were AC. The mean age was 60.31 ± 10.84 years (age range 33‒83 years). Within the AC group, 69 and 24 cases were low-grade and high-grade adenocarcinomas, respectively (**Figures 2**–**4**); 38 and 55 cases were pT1-2 stage and pT3-4 stage, respectively; 64 and 29 cases were pN0 stage and pN1-2 stage, respectively; 23 and 70 cases were positive and negative perineural invasion, respectively; 26 and 67 cases were positive and negative lymphovascular invasion, respectively; 25 and 68 cases were positive and negative EMVI, respectively; 90 and 3 cases were positive and negative CRM, respectively. Clinical features, histopathologic characteristics were summarized in **Table 2**.

**Table 2:** Clinical and pathological characteristics.

Category	Gender (Male/Female)	Relationship with peritoneal reflection (Above/Across/Below)	Tumor location (Upper/Middle/Lower)	Tumor longitudinal diameter (≥50mm/<50mm)	Tumor transverse diameter (≥10mm/<10mm)	Bowel circumferential involvement (≥1/2/<1/2)
Pathological type						
MC (n=17)	9/8	3/6/8	5/7/5	10/7	14/3	15/2
AC (n=93)	60/33	15/28/50	32/41/20	36/57	70/23	74/19
WHO grade(AC)						
G1 (n=2)	1/1	0/1/1	1/1/0	0/2	0/2	0/2
G2 (n=67)	46/21	10/18/39	13/41/13	22/45	50/17	57/10
G3 (n=24)	17/7	3/7/14	4/15/5	10/14	21/3	21/3
T stage(AC)						
pT1 (n=5)	3/2	0/0/5	0/2/3	2/3	3/2	3/2
pT2 (n=33)	25/8	3/8/22	4/24/5	17/16	25/8	24/9
pT3 (n=47)	32/15	11/13/23	14/19/14	16/31	39/8	42/5
pT4 (n=8)	3/5	0/5/3	2/4/2	5/3	7/1	8/0
N stage(AC)						
pN0 (n=64)	40/24	10/14/40	11/39/14	33/31	53/11	54/10
pN1 (n=17)	12/5	1/8/8	3/9/5	11/6	13/4	15/2
pN2 (n=12)	7/5	2/5/5	4/7/1	7/5	9/3	11/1
Perineural invasion(AC)						
Positive (n=23)	14/9	1/5/17	3/13/7	12/11	20/3	21/2
Negative (n=70)	49/21	12/21/37	15/41/14	35/35	52/18	58/12
Lymphovascular invasion(AC)						
Positive (n=26)	15/11	4/9/13	5/13/8	16/10	20/6	21/5
Negative (n=67)	47/20	10/17/40	13/42/12	33/34	53/14	57/10
EMVI(AC)						
Positive (n=25)	14/11	3/8/14	7/12/6	14/11	23/2	25/0
Negative (n=68)	49/19	10/18/40	10/43/15	33/35	51/17	54/14

MC, mucinous adenocarcinoma; AC, common adenocarcinoma.

### Interobserver Agreement

The intraclass correlation coefficient were 0.942 (95% CI 0.831–0.967) for APT SI; 0.862 (95% CI, 0.714–0.913) for D; 0.762 (95% CI, 0.632–0.825) for D*; 0.859 (95% CI, 0.697–0.912) for f; and 0.916 (95% CI, 0.850–0.933) for ADC, respectively. There were good agreements between two observers for measurements of APT SI, D, D*, f, and ADC values.

### Comparison of the Parameters in Different Groups of Rectal Adenocarcinomas

**Figure 5:**
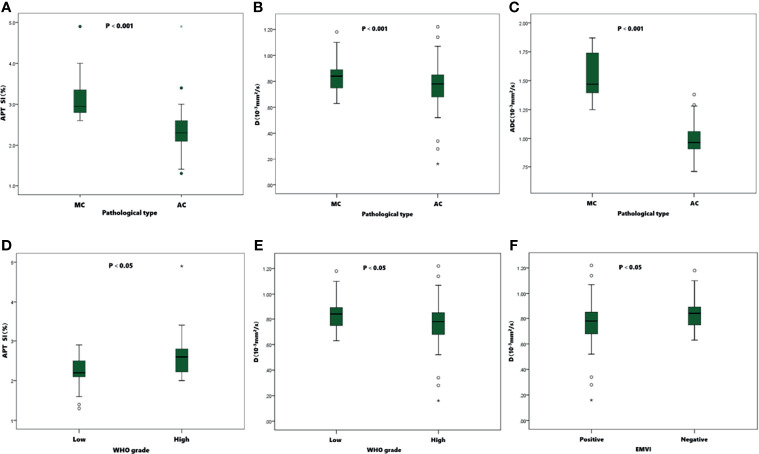
Boxplots of APT SI, D and ADC values in different pathological types, WHO grades, and EMVI statuses of tumors. The APT SIs **(A)**、D **(B)** and ADC **(C)** values were significantly higher in MC than in AC. In AC group, the APT SIs **(D)** were significantly lower in low-grade than in high-grade group, and the D values **(E)** were significantly higher than in high-grade group. The D values **(F)** were significantly lower in positive EMVI than negative EMVI.

Detailed results were showed in **Tables 3**, **4** and **Figure 5**. The APT SI, D and ADC values of MC were significantly higher than those of AC (all *P*<0.001). Within the AC group, the APT SIs were significantly lower, and the D values were higher in low-grade adenocarcinomas than in high-grade ones (*P*=0.001 and 0.025; respectively). The D values were significantly lower in positive than in negative EMVI tumors (*P*=0.045). No significant difference of APT SI, D, D*, f or ADC observed in other groups (all *P*>0.05).

**Table 3:** The comparison of APT SI, D, D*, f and ADC values in different groups of types.

Groups	APT SI (%)	D (×10^-3^mm^2^/s)	D* (×10^-3^mm^2^/s)	f	ADC (×10^-3^mm^2^/s)
Gross types					
Ulcerated (n=73)	2.578 ± 0.241	0.965 ± 0.217	6.307 ± 2.135	0.163 ± 0.068	0.956 ± 0.127
Elevated (n=37)	2.612 ± 0.325	0.942 ± 0.228	6.791 ± 2.139	0.184 ± 0.045	0.911 ± 0.130
*P* value	0.185	0.107	0.218	0.195	0.136
Histological types					
MC (n=17)	3.192 ± 0.661	1.153 ± 0.238	7.017 ± 2.579	0.150 ± 0.073	1.535 ± 0.203
AC (n=93)	2.333 ± 0.471	0.792 ± 0.173	6.989 ± 2.711	0.212 ± 0.033	0.986 ± 0.124
*P* value	0.000	0.000	0.267	0.106	0.000

ADC, apparent diffusion coefficient.

**Table 4:** The comparison of APT SI, D, D*, f and ADC values ​​in different groups of AC.

Groups	APT SI (%)	D (×10^-3^mm^2^/s)	D* (×10^-3^mm^2^/s)	f	ADC (×10^-3^mm^2^/s)
WHO grade					
Low-grade (n=69)	2.226 ± 0.347	0.842 ± 0.148	7.193 ± 2.913	0.225 ± 0.141	1.004 ± 0.129
High-grade (n=24)	2.668 ± 0.438	0.777 ± 0.178	6.361 ± 1.877	0.171 ± 0.100	0.929 ± 0.085
*P* value	0.001	0.025	0.420	0.124	0.155
T stage					
pT1-2 (n=38)	2.417 ± 0.318	0.772 ± 0.193	6.837 ± 2.502	0.214 ± 0.158	0.983 ± 0.129
pT3-4 (n=55)	2.276 ± 0.335	0.806 ± 0.158	7.094 ± 2.865	0.211 ± 0.116	0.989 ± 0.121
*P* value	0.399	0.447	0.778	0.775	0.795
N stage					
pN1-2 (n=29)	2.279 ± 0.366	0.770 ± 0.175	6.689 ± 2.015	0.189 ± 0.090	0.952 ± 0.110
pN0 (n=64)	2.236 ± 0.413	0.841 ± 0.160	7.127 ± 2.575	0.222 ± 0.149	1.002 ± 0.127
*P* value	0.692	0.157	0.163	0.359	0.074
Perineural invasion					
Positive (n=23)	2.325 ± 0.409	0.778 ± 0.186	5.986 ± 1.399	0.203 ± 0.085	0.946 ± 0.184
Negative (n=70)	2.335 ± 0.491	0.841 ± 0.111	7.294 ± 2.938	0.215 ± 0.145	0.998 ± 0.131
*P* value	0.609	0.098	0.064	0.830	0.123
Lymphovascular invasion					
Positive (n=26)	2.354 ± 0.443	0.787 ± 0.169	6.660 ± 2.092	0.201 ± 0.081	0.968 ± 0.101
Negative (n=67)	2.324 ± 0.485	0.805 ± 0.185	7.117 ± 2.921	0.217 ± 0.149	0.993 ± 0.131
*P* value	0.294	0.799	0.689	0.847	0.389
EMVI					
Positive (n=25)	2.416 ± 0.288	0.771 ± 0.175	6.929 ± 2.115	0.201 ± 0.114	0.979 ± 0.114
Negative (n=68)	2.306 ± 0.315	0.858 ± 0.151	7.009 ± 2.891	0.216 ± 0.140	0.988 ± 0.127
*P* value	0.139	0.045	0.665	0.654	0.901

### Comparison of ROC Curves for Distinguishing MC From AC, Low- From High-Grade AC, and Distinguishing EMVI Status

**Figure 6:**
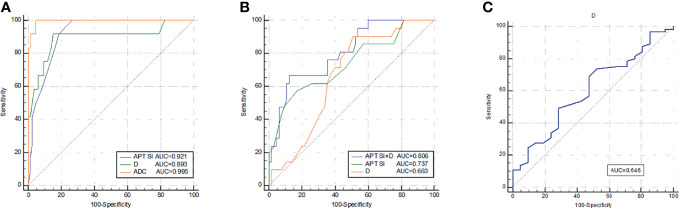
ROC curves of APT SI, D, and ADC for discrimination between MC and AC **(A)**; ROC curves of APT SI, D, and APT SI combined with D for discrimination between low- and high-grade AC **(B)**; and the ROC curve of D for discrimination between positive EMVI and negative EMVI **(C)**. All parameters were with significant differences between the groups.

ROC curves of APT SI, D, and ADC values between MC and AC, low- and high-grade AC, positive and negative EMVI were listed in **Figure 6**. The ROC curves for distinguishing MC from AC were shown in **Figure 6A** using the APT SI, D and ADC values with the AUCs of 0.921, 0.893, 0.995, respectively. The comparison among these AUCs showed no significant difference (APT SI *vs*. D: *Z*=0.352, *P*=0.725; APT SI *vs*. ADC: *Z*=2.457, *P*=0.140; and D *vs*. ADC: *Z*=1.607, *P*=0.108; respectively). The AUCs for distinguishing low- from high-grade AC using the APT SI and D values were 0.737 and 0.663, respectively (**Figure 6B**), without significant difference (*Z*=0.748, *P*=0.455). The AUC was increased to 0.806 through the combination of APT SI and D values (**Figure 6B**). The comparison of AUCs showed significant differences between the combined parameter and APT SI (*Z*=1.962, *P*=0.049) or D values (*Z*=2.040, *P*=0.041). The AUC for distinguishing positive EMVI from negative EMVI using the D value was 0.646 (**Figure 6C**). The diagnostic performance and optimal diagnostic threshold of parameters were listed in **Table 5**.

**Table 5:** Diagnostic performance of APT SI, D and ADC values with significant difference between groups.

Category	*P* value	AUC (95% CI)	Threshold	Sensitivity (%)	Specificity (%)
MC *vs*. AC					
APT SI	0.000	0.921(0.895-0.978)	2.650%	91.7	81.6
D	0.000	0.893(0.767-1.000)	0.930×10^-3^mm^2^/s	91.7	84.9
ADC	0.000	0.995(0.986-1.000)	1.230×10^-3^mm^2^/s	100	95.4
Low- *vs*. high grade AC					
APT SI	0.001	0.737 (0.607-0.867)	2.550%	57.1	81.8
D	0.025	0.663(0.544-0.782)	0.851×10^-3^mm^2^/s	48.5	80.5
APT SI + D	0.000	0.806(0.702-0.910)	N/A	76.7	87.7
EMVI (+) *vs*. (-) AC					
D	0.045	0.646(0.511-0.780)	0.785×10^-3^mm^2^/s	76.2	76.9

AUC, area under the curve; CI, confidence interval; APT SI +D, APT SI combined with D values.

## Discussion

The histopathologic type, tumor grade, T stage, N stage, perineural invasion, lymphovascular invasion, and EMVI statuses are important prognostic factors for rectal cancer. In our study, we performed a comprehensive investigation of correlations of APT and IVIM parameters with rectal cancer prognostic factors, in comparison with results by DWI. Results indicated that APT, IVIM, and DWI all can be used in differentiating between AC and MC. APT and IVIM can be used in differentiating grades of AC, and the combination of APT with IVIM could improve the diagnostic performance. DWI can’t be used in differentiating grades of AC.

We observed that APT SIs were significantly higher in MC than AC. According to the literature, APT SI was mainly contributed by the endogenous cellular proteins and peptides and affected by intercellular pH environment. Otherwise, cell density, mucin and angiogenesis also have significant effects on APT SI (17, 29). MC is characterized by tumor cell hypersecretion, with more than 50% of mucus content in the tumor parenchyma (5), which may have contributed to the higher APT SIs. D is the pure diffusion coefficient representing pure molecular diffusion, D* is the pseudo-diffusion coefficient representing microperfusion related diffusion, while f is the perfusion fraction related to microcirculation. Our study also found that D and ADC values of MC were significantly higher than those of AC, which was in accordance with previous research (30). Mucinous adenocarcinoma cells float on a layer of mucus in a relatively loose arrangement, which may decrease the cellularity and facilitate water molecule movement (31). The D* and f values showed no significant difference for distinguishing MC from AC, which may indicate the similar microperfusion component in these two types of lesions.

The histologic grade is an important prognostic factor for rectal adenocarcinoma. We demonstrated that the APT SIs of low-grade adenocarcinomas were significantly lower compared to those of high-grade adenocarcinomas, which was consistent with previous studies (25, 26). Therefore, APT weighted imaging may be helpful to identify the pathological grade of rectal cancer. Similar results have been reported in other tumors. For example, Sotirios et al. found that APT could differentiate low- from high-grade gliomas and predict the histopathological grade potentially (32). A study by Yin et al. demonstrated that APT SIs were significantly higher in prostate cancer than in benign prostatic hyperplasia and showed a strong correlation with the Gleason score (33). All these studies indicated that malignant tumors commonly have significantly higher APT SIs compared to those of the benign or normal tissues, and the APT SI tends to increase as the pathological grade advanced (34, 35). The higher APT SIs in high-grade tumors can be due to the abundant proteins production, rapid cell proliferation and angiogenesis. Previous studies suggested that IVIM was helpful to assess tumor grades of intracranial tumors, solid soft-tissue tumors, HCC, and prostate cancer etc., and the D value was observed to be inversely correlated with the tumor grade (36–39). In present study, lower D values were observed in high-grade than those of low-grade rectal adenocarcinomas, which was in agreement with previous results (11, 40, 41). The D value, that represents the pure diffusion of free water molecules, was decreased with the increasing cellularity, tight cellular structure in high-grade tumors. The AUCs of APT and D for distinguishing low- from high-grade adenocarcinomas were 0.737 and 0.663 respectively, with moderate diagnostic performance. The AUC was raised to 0.806 by the combination of APT SI and D values, with 76.7% sensitivity and 87.7% specificity. The D* and f values showed a trend of decreasing with increased tumor grades in our study but without statistically significance. Furthermore, previous studies showed D* or f was negative correlated with tumor grade in rectal cancer (11, 30, 40, 41). A possible explanation is that tumor cells grow rapidly in high-grade tumor, leading to immature vascular structure and reduced microcirculation perfusion thus lower perfusion-related parameters, such as D* and f values. The ADC values showed no significant differences for distinguishing tumor grade of AC in present study, which may be caused by the integrated effects of both diffusion and microperfusion.

Tumor stage is closely related to prognosis for rectal adenocarcinoma. In present study, postoperative pathological stage was used to retrospectively analyze the correlation of APT and IVIM parameters with tumor stages. Parameters derived from APT and IVIM showed no significant differences between pT1-2 and pT3-4 stages, or between pN1-2 and pN0 stages. These results were inconsistent with previous studies which showed that APT SIs were higher in advanced T stage and lymph node metastasis (25, 26). However, T1 or T4 stage cases were absent in previous studies, and the positive rate of lymph node metastasis was higher in previous studies than our study, which may cause the selection bias of the sample. Sun et al. observed that D and D* showed a trend of decreasing with the increasing of tumor clinical stages and lymph node metastasis in rectal cancer (40). The parameters derived from APT and IVIM might exhibit more aggressive biologic behavior, further study is needed to evaluate the significance.

EMVI refers to the presence of tumor infiltration in the vessels outside the muscularis propria, and it is an independent prognostic factor of rectal cancer. Positive EMVI exhibits more local recurrence, more distant metastasis, and more tumor-related death (42). Although Chen et al. suggested that APT SIs were higher in EMVI-positive than in EMVI-negative cases (26), our study showed no significant difference of APT SI in EMVI involvement. We considered that the inconsistent results might be related to the different positive rate of EMVI status (26.9% in present study while 50.8% in the previous study). In addition, the D value was observed to be lower in the positive EMVI group than in the negative group in this study, while Wei et al. identified that D value was lower in microvascular invasion (MVI)-positive than in MVI-negative HCCs (43). The decreased D value may be because tumor emboli or clusters of cancer cells restrict the diffusion of water molecules. The AUC for distinguishing EMVI involvement using the D value was 0.646 with moderate diagnostic significance. The high-resolution T_2_WI images should be combined to improve the diagnosis accuracy of EMVI, which was considered positive if vessel wall irregularity, abnormal extension, suspected the empty signal was replaced by tumor tissue with intermediate signal intensity. Additionally, perineural invasion and lymphovascular invasion are prognostic factors for rectal cancer associated with recurrence, metastasis, and postoperative adjuvant therapy. No significant difference of parameters derived from APT and IVIM were found in groups with and without different types of structure invasion in our study, which may be because the tumor microenvironment reflected by APT or IVIM parameters is insufficient to cause significant changes in perineural and lymphovascular invasion.

The present study has some limitations. First, patients with locally advanced rectal cancer received neoadjuvant therapy were excluded, potentially causing selection bias. Second, only 2 types of rectal adenocarcinomas were collected, and further studies with abundant cases are needed to be explored. Third, the choice of different ROIs may also lead to differences in results due to tumor heterogeneity. Furthermore, this study did not analyze the correlation of APT and IVIM parameters with immuno-histochemical indicators or gene expression. In the future, collection of complete data for more in-depth research is needed.

## Conclusion

APT SI and D values can be used in discriminating between MC and AC, slightly inferior to ADC. The APT SI and D values were helpful to differentiate the low- and high-grade of AC, and the combination of APT SI with D values could improve the diagnostic performance. The D value can help determine EMVI status for AC patients. However, it is still debatable whether APT or IVIM can help distinguish stage, perineural invasion, and lymphovascular invasion. In conclusion, APT and IVIM were helpful to assess the prognostic factors related to rectal adenocarcinoma, including histopathological type, tumor grade and EMVI status.

## Data Availability Statement

The raw data supporting the conclusions of this article will be made available by the authors, without undue reservation.

## Ethics Statement

Written informed consent was obtained from the individual(s) for the publication of any potentially identifiable images or data included in this article.

## Author Contributions

JL: manuscript preparation, literature research, data acquisition, statistical analysis, and manuscript editing. LL: study design, manuscript preparation, data analysis, and manuscript revising. XG: manuscript preparation and literature research. SL and JC: study conception and design, manuscript review, and guarantor of integrity of the entire study. All authors contributed to the article and approved the submitted version.

## Conflict of Interest

Author LL was employed by Philips Healthcare.

The remaining authors declare that the research was conducted in the absence of any commercial or financial relationships that could be construed as a potential conflict of interest.

## Publisher’s Note

All claims expressed in this article are solely those of the authors and do not necessarily represent those of their affiliated organizations, or those of the publisher, the editors and the reviewers. Any product that may be evaluated in this article, or claim that may be made by its manufacturer, is not guaranteed or endorsed by the publisher.
